# A Tailored Sequencing Approach for Identifying SARS-CoV-2 Variants in Challenging Samples from Mink

**DOI:** 10.3390/ijms27219638

**Published:** 2026-10-28

**Authors:** Aleksandra Giza, Ewelina Iwan, Arkadiusz Bomba, Anna Orłowska, Marcin Smreczak, Marta Pietruk, Aleksandra Chudzik, Małgorzata Olejnik, Katarzyna Domańska-Blicharz

**Affiliations:** 1 Department of Research Support, National Veterinary Research Institute (PIWet-PIB), 24-100 Puławy, Poland; ewelina.iwan@piwet.pulawy.pl (E.I.); arkadiusz.bomba@piwet.pulawy.pl (A.B.); marta.pietruk@piwet.pulawy.pl (M.P.); aleksandra.chudzik@piwet.pulawy.pl (A.C.); molejnik@umk.pl (M.O.); 2 Department of Virology and Viral Animal Diseases, National Veterinary Research Institute (PIWet-PIB), 24-100 Puławy, Poland; anna.orlowska@piwet.pulawy.pl (A.O.); smreczak@piwet.pulawy.pl (M.S.); katarzyna.domanska@piwet.pulawy.pl (K.D.-B.); 3 Department of Basic and Preclinical Sciences, Faculty of Biological and Veterinary Sciences, University of Nicolaus Copernicus in Toruń, 87-100 Toruń, Poland

**Keywords:** viral sequencing, metagenomics, hybrid capture, library preparation, SARS-CoV-2, epidemiological surveillance, mink

## Abstract

High-throughput sequencing (HTS) is an essential tool for genomic surveillance of infectious agents, including SARS-CoV-2 in farmed mink. However, sample quality varies, hindering high-quality sequencing results. In this study, six mink-origin SARS-CoV-2-positive samples were sequenced using two hybrid-capture methods (commercial and custom) and a shotgun metagenomic approach. All methods consistently identified SARS-CoV-2 variants. The metagenomic approach yielded the lowest mapping rate (up to 0.35%), breadth of coverage (97–100%), depth of coverage (9–932×), and mean mapq (30–59), and one sequence did not meet the quality criteria. In contrast, both hybrid-capture methods achieved higher mapping rates (47–99%), breadth of coverage (99.9–100%), and depth of coverage (508–115,846×). The custom hybrid-capture approach was selected because of its superior coverage uniformity, the lowest number of ambiguous bases (N), adjustability to low RNA levels and degraded material, and streamlined laboratory workflow. To further evaluate its applicability, it was applied to 35 clinical samples collected as part of routine genomic surveillance. This method generated sequencing data with high mapping rates (median 95%), depth of coverage (median 13,238×), breadth of coverage (median 100%), and mean mapq (median 60). These findings demonstrate that the custom hybrid-capture approach is a useful tool for genomic surveillance of SARS-CoV-2 in mink-origin samples.

## 1. Introduction

Besides humans, SARS-CoV-2 can infect various animals, including cats, dogs, ferrets, hamsters, gorillas, lions, snow leopards, hippopotamuses, white-tailed deer, and American mink [1,2]. American mink and ferrets are highly susceptible to SARS-CoV-2, and their high-density co-housing at fur farms promotes the risk of virus transmission among the animals. Many mink were infected within a short period of time, and an increase in prevalence from 4% to 97% in eight days among mink on a farm in Denmark was reported [3]. As of January 2021, SARS-CoV-2 has been detected in numerous mink farms for fur production in different countries of the EU, including Denmark, France, Greece, Italy, Lithuania, The Netherlands, Spain, and Sweden. Sporadic SARS-CoV-2 cases have also been recorded in kept ferrets in Slovenia and Poland [4,5]. Due to the open design of mink farms, stray and wild animals may encounter mink and their excreta, potentially carrying infections outside the farm.

Several studies conducted at mink farms reported an average of two mutations per month, which may increase the risk of animal-to-human transmission [6,7]. The virus introduced to mink by infected humans, particularly mink keepers, can spread among mink and zoonotically back to humans or infect other animals in a farm setting [5,7]. Therefore, SARS-CoV-2 on mink farms poses a potential risk of mutation development and the emergence of new variants through passaging in new hosts or even recombination with species-specific coronaviruses [1,5]. In January 2021, the EU recommended surveying SARS-CoV-2 variants on mustelids, particularly mink and raccoon dogs [8]. In line with international recommendations [9,10], viruses were categorized into three groups based on their potential impact on spread, disease severity, diagnostics, therapeutics, and vaccines: Variants Under Monitoring (VUM), Variants of Interest (VOI), and Variants of Concern (VOC). This classification alerts member countries to new SARS-CoV-2 variants that could disrupt the epidemiological situation [9,10]. Based on the European Centre for Disease Prevention and Control (ECDC) and the World Health Organization (WHO) recommendations, countries should remain alert for signs of new SARS-CoV-2 lineages or variants and have implemented methods to identify VOCs, VOIs, or VUMs through WGS-based genomic monitoring [11]. The SARS-CoV-2 monitoring system for mink implemented in Poland resulted in the identification of 18 positive farms between January 2021 and May 2023. At the same time the virus was detected, attempts were undertaken to sequence the whole of the viral genome.

Determining complete viral sequences is essential for monitoring, as it enables the discovery of phylogenetic relationships, transmission pathways, evolutionary dynamics, outbreak patterns, and risk assessment [11,12,13]. It also supports the design and updating of genetic viral identification assays, the development of vaccines, and the design and evaluation of antiviral drugs [14]. High-throughput sequencing (HTS) is the primary method for identifying SARS-CoV-2 variants, with various approaches.

Shotgun metagenomics is the least biased sequencing method [15]. It is useful for characterizing the entire genetic makeup of a sample, even for viruses that have not been previously identified. However, this technique generates a large volume of data at a high cost. Additionally, limited sensitivity may yield low-depth or incomplete genomes from samples with low viral loads [15]. Consequently, depletion methods or targeted approaches are used to enrich the SARS-CoV-2 genome. Depletion involves filtration, centrifugation, or enzymatic/chemical removal of unwanted sequences (e.g., the host) [13]. Targeted metagenomics involves enriching the sample to select only viral genetic material, which can be achieved by hybridization with probes complementary to viral sequences or by amplification with specifically designed primers. While targeted metagenomics is less costly and more sensitive than shotgun metagenomics, it requires prior knowledge of the virus’s sequence and the development of specific sequencing methods [13].

The amplicon-based approach is affordable and requires less time to design primers and implement in the lab [13]. However, viruses continue to evolve, and mutations in the primer-binding site can impact amplification, necessitating primer redesigns. PCR-based methods are also more prone to contamination and bias [13,15,16] and face challenges in covering the ends of the SARS-CoV-2 genome [15,17]. Probe-based methods are more adaptable to different virus variants, tolerating up to 10–20% [13] or even 20–30% [18] differences between the probes and the tested sequence. The probe-based approach offers high sensitivity [13] and consistent depth of coverage across the genome at various Ct values, unlike the uneven coverage seen with amplicon-based and shotgun metagenomic methods [15]. However, the main limitations of probe-based methods are their reliance on the quality of library preparation and proper probe design for efficiency and specificity [19]. Additionally, they incur higher costs, longer workflow times, and less throughput than the amplicon-based approach [20].

Obtaining a high-quality SARS-CoV-2 genome sequence from routinely collected mink swab samples is difficult. Animals within the same herd differ in their infection stage; thus, their throat swabs may contain little viral genetic material [1]. Additionally, these samples may contain a range of substances that induce degradation of genetic material; pre-analytical conditions also play a huge role in sample stability [21,22]. The performance of SARS-CoV-2 sequencing approaches may also be influenced by the background nucleic acid content and microbial composition of mink oral swabs. Host-derived and non-target microbial sequences may reduce the proportion of sequencing reads available for viral genome reconstruction. Although NGS-based approaches for SARS-CoV-2 genomic surveillance have been extensively evaluated using human clinical specimens [15,16,17], the genetic background in mink oral samples remains poorly characterized. Therefore, sample-specific methodological optimization is required, and the main challenges are the limited quantity and poor quality of available mink oral swab material.

This study provides a comparative evaluation of two probe-based methods: custom (HC Custom) and commercial approach (HC Commercial), as well as shotgun metagenomics (S-MG), for whole-genome sequencing of SARS-CoV-2 identified in mink farms in Poland. Given the variable quality and quantity of mink-derived material, the HC Custom approach incorporates modifications designed to enable the adaptation of library preparation parameters to the characteristics of individual samples. This study further identifies the most robust methodology for processing challenging SARS-CoV-2-infected mink samples and demonstrates the applicability of the optimized custom approach using clinical material from genomic surveillance.

## 2. Results

### 2.1. The Comparative Study

For a comparative study, six samples (S1–S6) (Table 1, and Supplementary Table S1) were prepared using HC Commercial, HC Custom, and S-MG library preparation approaches. Eighteen whole-genome sequences were generated, and one S-MG-prepared sequence (211123_5130) did not meet quality standards. Raw sequencing data have been deposited in the NCBI Sequence Read Archive (SRA) under BioProject accession number PRJNA1462178.

**Figure 1:**
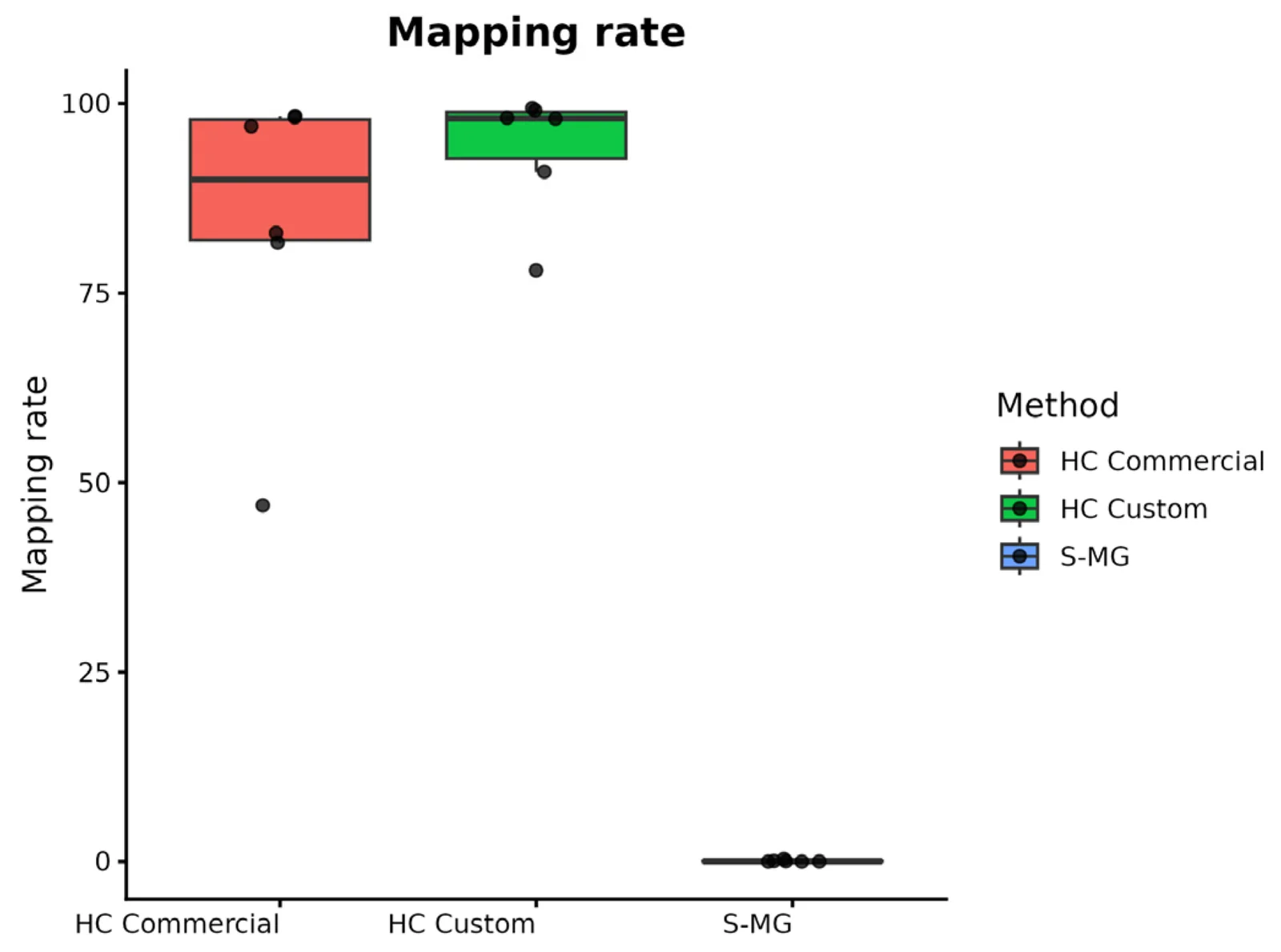
Boxplot of mapping rates obtained using the three tested methods.

The mapping rate in the S-MG approach was the lowest (up to 0.35%). In contrast, both enriched methods showed higher mapping rates than the shotgun metagenomic approach—HC Commercial ranged from 46.9% to 98.3%, and HC Custom from 78.5% to 99.4%. The lowest mapping rate for each method was observed in sample S2 (Table 1), which contained a relatively low amount of SARS-CoV-2 and presented the highest Ct value. Differences among the three methods were statistically significant (*p* = 0.006). However, pairwise comparisons showed that although the custom method produced better results than HC Commercial, these differences were not statistically significant (*p* = 0.156) (Figure 1). Detailed statistical analyses are provided in Supplementary Table S3.

**Figure 2:**
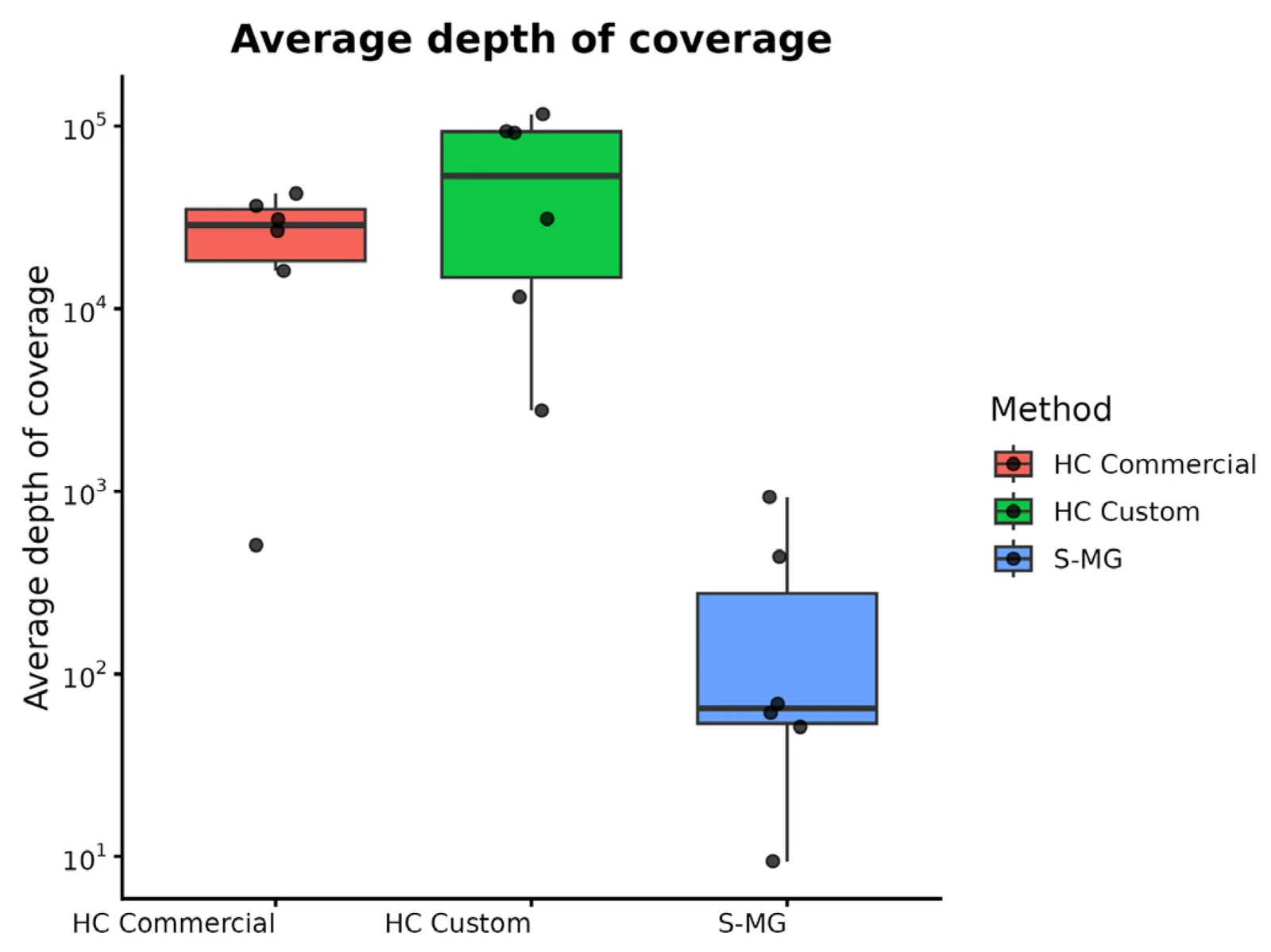
Boxplot of average depth of coverage obtained using the three tested methods.

The average depth of coverage varied across methods (Table 1). In the metagenomic approach, the range was 9× to 932×. In both hybrid capture methods, the viral genome was obtained at significantly higher average depth of coverage (508×–42,736× for HC Commercial and 2771×–115,846× for HC Custom). Differences between metagenomic and enrichment-based methods were statistically significant (*p* = 0.009). The mean average depth of coverage was higher for the HC Custom (57,766.83×) approach than for the HC Commercial (25,565.17×); however, this difference was not statistically significant (*p* = 0.156) (Figure 2).

**Figure 3:**
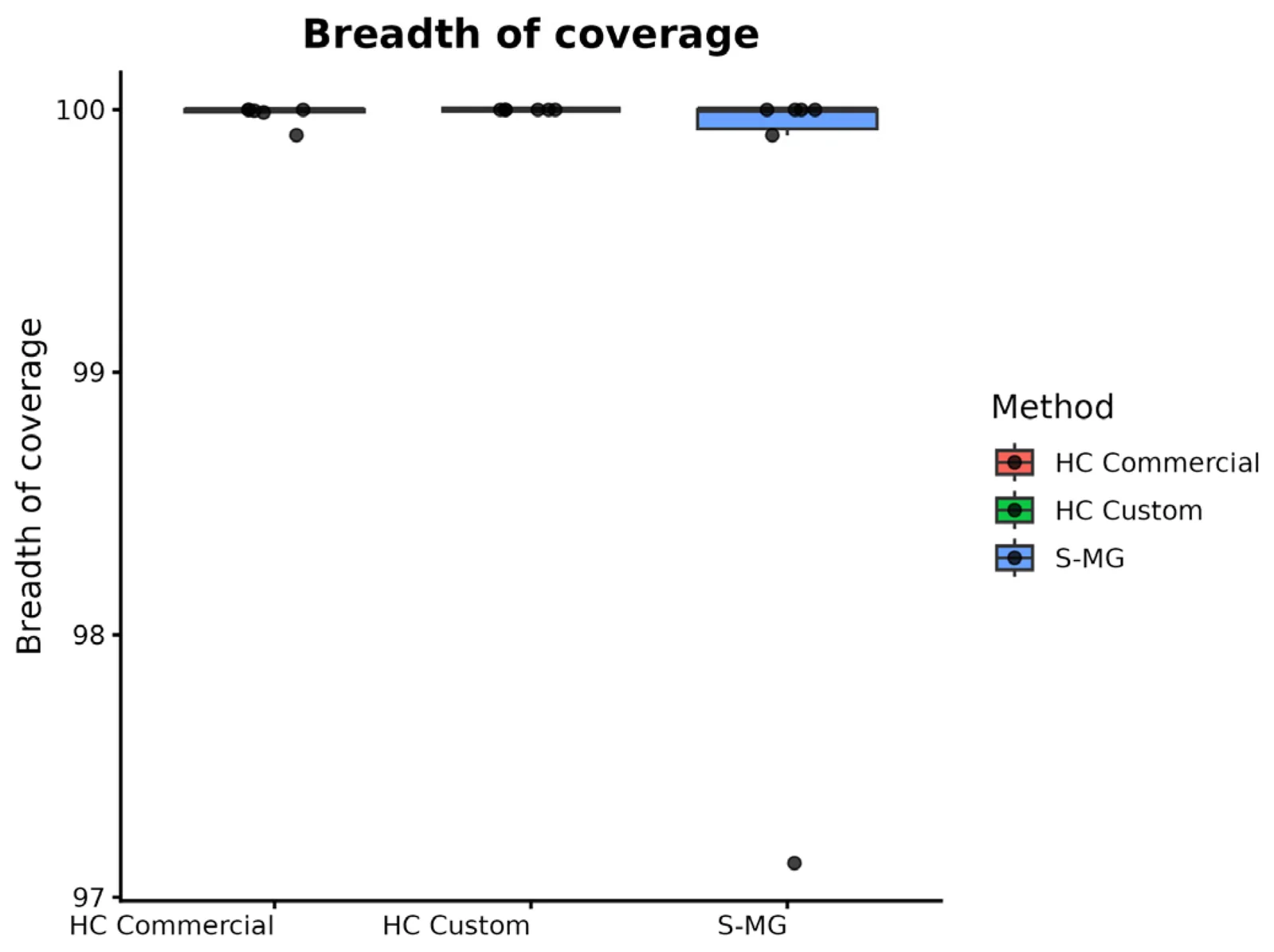
Boxplot of the breadth of coverage obtained using the three tested methods.

Additionally, no statistically significant differences in breadth of coverage (Table 1) were observed among the three tested methods (*p* = 0.1778) (Figure 3).

**Figure 4:**
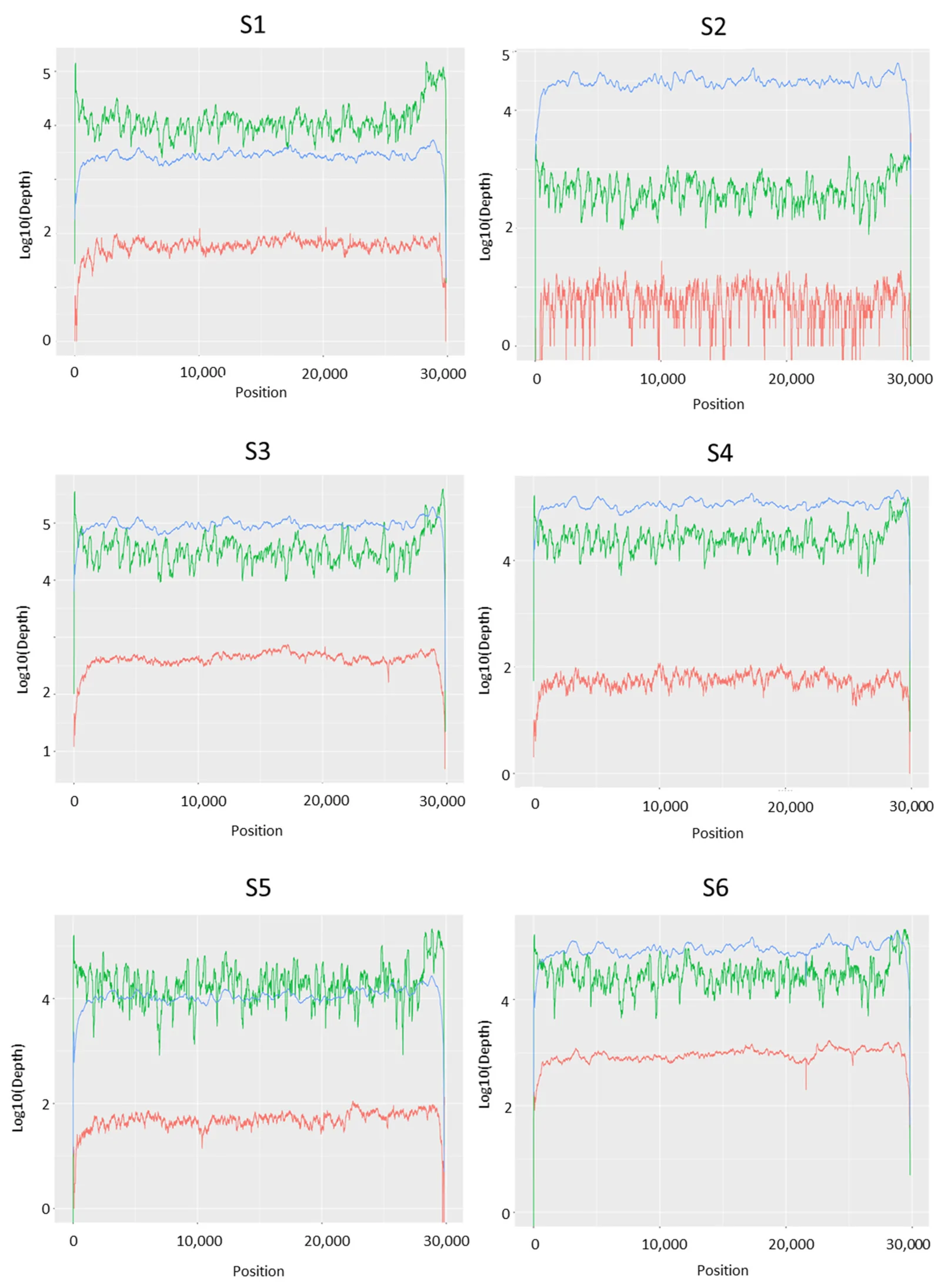
The figure shows the depth of coverage (log10(Depth)) across the SARS-CoV-2 genome (Position), obtained with the tested methods. The red line represents the depth of coverage of the S-MG method, the green line shows HC Commercial, and the blue line shows HC Custom.

To better visualize the breadth and uniformity of coverage, sequence gaps, and low-depth regions, the coverage charts were analyzed (Figure 4). Complete viral genomes were obtained in 17 of the 18 sequencing datasets. In sample S2 (Table 1, Figure 4), prepared with the S-MG approach, the SARS-CoV-2 genome was not fully covered, with gaps across the sequence and low-depth regions; therefore, it was excluded from subsequent comparisons. Ambiguous nucleotides (N) were observed at the ends of some generated sequences. This issue occurred in every S-MG-prepared sample (2–335 ambiguous nucleotides), in four HC Commercial-prepared samples (2–23 N), and in one HC Custom-prepared sample (10 N). Across unambiguous positions, both hybrid capture methods showed 100% sequence identity, and compared to S-MG, the identity was 99.98%. Based on the coverage charts, HC custom coverage was the most uniform.

The lowest mean mapq (30.3–59.3) was observed with the shotgun metagenomic approach. In both hybrid capture methods, mapq values were close to 60 (59.9–60).

Both clade and Pango lineages were the same across methods.

All three approaches enable whole-genome SARS-CoV-2 sequencing; however, S-MG produced the lowest sequence quality. HC Custom tended to generate higher-quality sequences than HC Commercial; however, differences in mapping rate, depth, and breadth of coverage were not statistically significant. HC Custom showed the most uniform coverage and the fewest ambiguous bases, and yielded the best sequencing results for the S2 sample, which had the lowest viral content.

### 2.2. The Comparison of Laboratory Workflow and Practical Aspects of Tested Methods

S-MG is less efficient than both hybrid-capture methods. Although HC Custom and HC Commercial did not differ statistically, the results suggest that HC Custom could be more useful for challenging samples (like Sample S2 (Table 1)). Therefore, we also compared the methods in terms of their practical applicability in the laboratory. The comparison of practical aspects and efficiency of S-MG, HC Commercial, and HC Custom is presented in Table 2.

The S-MG laboratory protocol takes the least time compared with hybrid-capture methods because it omits the hybridization step, which requires time, the highest precision, and greater technical expertise. However, bioinformatic analysis of metagenomic data can be more complex because viral reads must be identified within a large background of host and other microbial sequences, whereas hybrid capture enriches the target viral sequences before sequencing. Total hands-on time for both hybrid-capture methods depends on the number of samples and the number of pools prepared simultaneously for hybridization. The HC Custom workflow requires 2–3 additional hours of hands-on time compared to HC Commercial due to the inclusion of extra quality control steps, while in the HC Commercial workflow, quality control is performed only at the final stage, immediately before hybridization. Quality control of the RNA before library preparation is not useful, because some mink-origin samples were below the quality required to start the HC Commercial protocol. The lack of a quality control step in HC Commercial means that if a library fails to meet the required quality criteria, it is difficult to identify the step responsible for the failure. Moreover, this can lead to substantial reagent consumption and considerable time and effort before the problem is detected. HC Custom adds an additional dsDNA quality-control checkpoint after reverse transcription, which serves as a starting point for subsequent library-preparation steps and increases the efficiency of later reactions. The HC Custom method includes a modification in the RNA reverse transcription step by multiplying the reaction volume, increasing the yield of RT and the input of cDNA for further analyses. Additionally, the method was optimized for three different DNA inputs. Finally, for highly degraded samples, the fragmentation times were adjusted.

The costs of every method are also worth considering. S-MG costs more than hybrid-capture methods because it requires higher sequencing throughput. The costs and required technical expertise for the two hybrid-capture approaches are similar.

The sensitivity of both methods is also crucial. With the HC Commercial method, SARS-CoV-2 was successfully sequenced at Ct 28, but not at 29–32. However, clinical samples presented lower RNA levels than recommended in the protocol (min. 50 ng in 10 µL), including below detection, and the RNA was of poor quality. In HC Custom, the protocol can be modified for samples with lower viral load (up to Ct 33.9) or degraded genetic material (the lowest tested average sample fragment size at 1153 bp or an invisible smear in the electropherogram).

To summarize, HC Custom has a tendency to yield higher-quality sequencing data, including more uniform coverage and fewer ambiguous bases (Section 2.1). However, quality parameters did not differ significantly. Adjusting library-preparation parameters to individual sample characteristics and adding an additional quality control step enabled high-quality sequencing data from degraded samples with low viral loads, highlighting the adaptability of the HC Custom approach. The costs of both methods are comparable. Due to a number of low-quality clinical samples, despite the longer hands-on time required for HC Custom, we selected it for the implementation study.

### 2.3. The Implementation Study of the Custom Method

35 samples from mink farm surveillance monitoring were processed using the HC Custom method during the implementation study to assess the validity of the new approach. The raw sequencing data from 35 sequences have been deposited in the NCBI Sequence Read Archive (SRA) under BioProject accession number PRJNA1462178. Ct values of samples ranged from 17.6 to 33.9. Sequencing of SARS-CoV-2-positive samples with higher Ct values was unsuccessful. The lowest tested average sample fragment size was 1153 bp, and we obtained high-quality whole-genome viral sequence. The average depth of coverage for SARS-CoV-2 in sequenced samples ranged from 335× to 113,363×, and the breadth of coverage ranged from 99.71% to 100%. The median of the mapping rate was 95%. The meanmapq value ranged from 57.1 to 60, with a median of 60 (Supplementary Table S2).

The implemented approach enabled the recovery of the complete SARS-CoV-2 genome sequence even from two samples with a very low amount of viral RNA (Ct values as high as 33.5–33.9). The breadth of coverage exceeded 99.99%, with an average depth of 59,065× (sample 34) and 335× (sample 35). The mapping rate varied—sample 34 showed 95.59%, comparable to or even higher than that of samples with higher virus RNA load (i.e., samples 3 and 8 with Ct values of 17.9 and 19.5, respectively). Conversely, sample 35 had a lower mapping rate of 2.1% and a slightly lower mean mapq of 57.1 (Supplementary Table S2).

**Figure 5:**
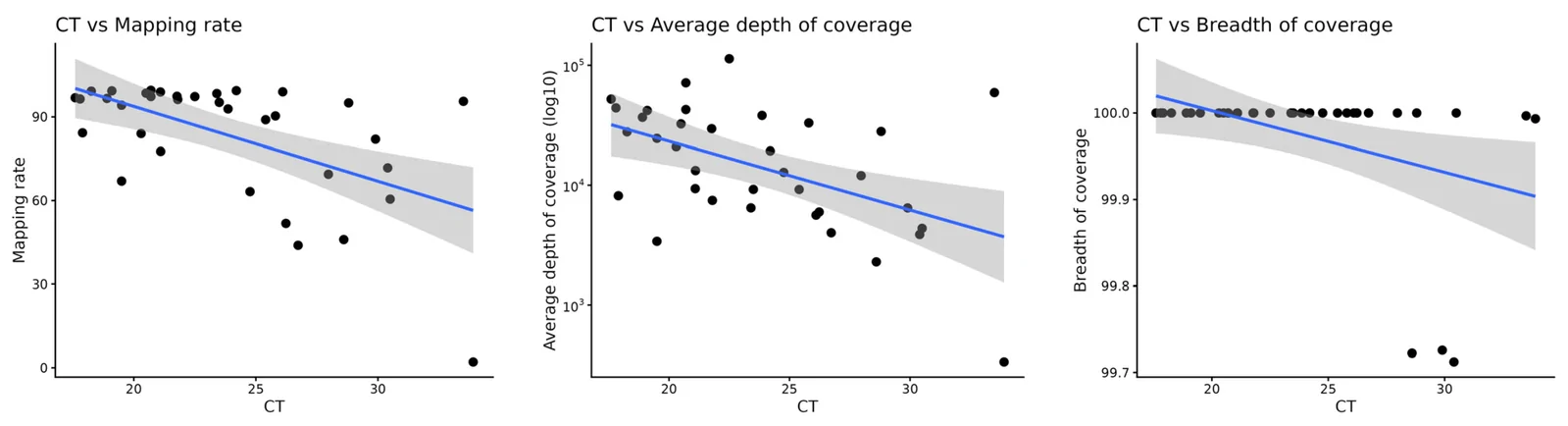
The dependence of the mapping rate/average depth of coverage/breadth of coverage on the Ct value for HC Custom.

The mapping rate, breadth of coverage, and average depth of coverage were affected by the viral load in the sample (Ct value) (Figure 5). Mapping rate and breadth of coverage showed a strong negative correlation, and average depth showed a moderate negative correlation (Supplementary Table S4).

These findings confirm the high quality of the genomic data obtained and the suitability of the HC Custom method for genomic surveillance of SARS-CoV-2. The implementation study also confirmed that HC Custom is more sensitive than HC Commercial.

## 3. Discussion

The WHO recommendations for genomic sequencing and analysis of SARS-CoV-2 [13] specify that sufficient high-quality SARS-CoV-2 RNA is necessary to maximize sequencing yield and the quality of genomic data. The issue of obtaining high-quality sequencing from low-quality material was raised by Lam et al. [23], who noted that suboptimal processes are common in high-throughput and centralized testing, affecting the quality of the available material. This issue served as the starting point for our research. The poor quality of mink-origin genomic material posed a challenge, requiring efforts to select an appropriate and effective sequencing approach.

S-MG was the first approach tested, but it has some limitations, including low sensitivity and high cost. The results of our study showed that one metagenomic sequence failed to meet quality standards, but variant identification was consistent across all samples prepared with different methods. The lowest mapping rates and depths for S-MG samples likely resulted from the high content of non-SARS-CoV-2 genetic material in the throat swabs. Similar results were obtained by Liu et al. [24] using three different metagenomic approaches on patient nasopharyngeal swab samples, with a mapping rate of up to 5.7% (higher in samples with higher viral input), which was much lower than that of the amplicon approach. Our study also indicated that shotgun metagenomics has limited utility for low-viral samples, as full viral sequences and high depth were achieved only in samples with a Ct value below 21. Similar findings were reported by Charre et al., who recommended S-MG only for samples with high viral loads [15]. The high cost of S-MG analysis further restricts its routine diagnostic use. Another issue was that ambiguous nucleotides (N) occurred at the ends of sequences in every S-MG-prepared sample, likely due to decreased sequencing coverage or reduced base-calling confidence in these regions. In view of these unsatisfactory results, and bearing in mind the need to obtain a complete set of molecular characteristics of the virus as quickly as possible during the COVID-19 pandemic, in the next stage we tested methods that would help to address these shortcomings, i.e., enrichment methods.

In the early stages of the COVID-19 pandemic, technical guidelines for SARS-CoV-2 genome sequencing [12] primarily recommended amplicon-based methods. However, the limitations of the amplicon-based approach, such as the need to redesign primers for newly emerging variants, susceptibility to contamination and bias, and issues with covering the ends of the SARS-CoV-2 genome [13,15,16,17], were reasons why we decided to implement a probe-based approach, which was also a part of the ECDC recommendations. Admittedly, Daviña-Núñez et al. [25] reported that the amplicon-based method had greater depth of coverage than the probe-based method, but the breadth of coverage of the whole genome was lower and varied depending on the variant, which affected the proper identification of the Delta variant [25]. Uncovering the spike protein region at specific residues can lead to variant assessment based on other characteristic mutations, potentially causing mismatches in variant identification, as seen in the commonly used ARTIC v4 protocol (residues 417, 440, 446) [11,19]. The ARTIC protocols are still being updated to reflect emerging viral variants, requiring ongoing revisions to laboratory protocols [11,15,23]. Issues with variant calling due to low breadth of coverage or sequence gaps have also been reported by Gerber et al. [17]. Rehn et al. [19], using the amplicon method, observed primer incompatibility in regions harboring mutations in new virus variants, in contrast to the probe-based method.

A major advantage of amplicon-based methods is their higher sensitivity compared to shotgun metagenomic and probe-based approaches, even for samples with Ct values up to 33.9, supporting high-throughput processing for large-scale surveillance [15,17]. However, monitoring mink farms involves substantially fewer samples than human surveillance, allowing more resources to be devoted to each individual sample. Moreover, we successfully sequenced two samples with Ct values above 33, prepared using HC Custom, demonstrating that the probe-based approach can work for low-viral samples without issues such as low-coverage regions, variant misidentification, or the need for protocol updates. Additionally, an overlapping probe design, as used in our panel, can improve hybridization efficiency and facilitate target capture in polymorphic regions and from highly fragmented DNA [26]. However, we did not perform parallel amplicon sequencing experiments; thus, this study cannot directly compare the performance of our probe capture methods and amplicon schemes at the experimental level.

The mapping rates in probe-based methods may depend on various factors, such as the amount of viral genome in the sample (Ct value), the laboratory enrichment procedure, the use of matched probes, and RNA quality. Our results obtained using the HC Custom method showed a significant correlation between the mapping rate and Ct, consistent with the results of Daviña-Nuñez et al. [25], who also used the Roche probe-based protocol. In turn, in the probe-based SureSelect XT HS2 RNA System (Agilent Technologies, Santa Clara, CA, USA) protocol, an experiment using 85 human nasopharyngeal swab samples, with uniformly low Ct values (Ct < 20), showed an average mapping rate of only 19.9% to the SARS-CoV-2 genome [17]. In both of our tested probe-based protocols, the mapping rates were considerably higher, even in samples with Ct values above 28. It is worth noting that a high-quality, fully covered genome was obtained using our HC Custom method even for samples with Ct values above 33. The probable reason for these results is a more closely matched probe panel and the high quality of the enrichment process in our methods. Charre et al. [15] tested the RVOP probe-based panel (Illumina, San Diego, CA, USA), which demonstrated high sensitivity for detecting long deletions but was not effective on samples with Ct values above 25 due to low depth and breadth of coverage [15]. In turn, Wen et al. [27], using self-designed probes, obtained complete SARS-CoV-2 genomes with coverage 14×–2370× from human nasopharyngeal swab samples with Ct values ranging from 32.36 to 38.44, despite very low mapping rates (<1%), whereas the upper Ct limit for successful genome recovery in our study was 33.9. However, the research was conducted on a small group of three samples using MGI sequencing technology [27]; therefore, it is difficult to compare with our results. In our study, based on coverage charts, HC Custom provided the most uniform breadth of coverage among the three tested methods, with high depth values across the entire sequence. In HC Custom, additional safe stopping points were introduced compared to HC Commercial, making the method more convenient (Section 4). Additional modifications compared to S-MG or HC-Commercial, such as adjustments to three input values, the number of PCR cycles, and fragmentation time based on sample amount and quality (Section 4), enabled successful library preparation across samples of varying quality. It is also important that probe-based methods are more cost-effective compared to S-MG.

Regardless of the HC method, average depth of coverage exhibited high variability among samples, a finding also supported by the implementation study and research by Daviña-Nuñez et al. [25]. In probe-based methods, accurate normalization before sequencing is difficult because hybridization efficiency varies between samples within a pool, disrupting the normalization process that occurs before hybridization [25]. Pooling samples with similar Ct values for hybridization in our research did not yield similar sequencing depths for the pooled samples, as expected. Therefore, sample-specific hybridization efficiencies may have introduced a potential confounding effect on sequencing depth, despite Ct-based grouping. Consequently, the contribution of pooling-related variability to the observed differences between samples and hybrid-capture approaches cannot be entirely excluded. However, this limitation did not affect the overall quality of the sequence data or the proper identification of variants.

Previous studies have shown that more PCR cycles may increase polymerase errors, potentially affecting sequence accuracy and variant identification [16,28,29]. The HC Commercial uses 19 PCR cycles during library preparation, compared with 8–10 in HC Custom. Despite this difference, the consensus sequences were identical at all unambiguous positions. However, this does not rule out differences in low-frequency variant detection due to PCR-induced errors or sequence filtering. As our study focused on identifying predominant SARS-CoV-2 variants, including VOCs, rather than within-sample diversity, the ability to detect low-frequency variants was not evaluated and remains a study limitation.

Despite previously demonstrated limitations, all methods similarly identified viral variants. The presented methods were used for genomic surveillance of SARS-CoV-2 in farmed mink in Poland between 2021 and 2023. It enabled the identification of eight different Pango lineages: 20B (two farms, B.1.1), Delta (21J, eight farms, AY.4, AY.43, AY.122, AY.126, B.1.617.2), Alpha (20I, one farm, B.1.1.7) and Omicron (21L, one farm, BA.2, 22B, one farm, BA.5.2) (Supplementary Tables S1 and S2). The vast majority of mink SARS-CoV-2 infections were traced back to variants dominant in human samples during the COVID-19 epidemic in Poland, although one of the Alpha variants was detected many months after the epidemic’s peak, suggesting their cryptic circulation in an unknown reservoir [1,5,8].

## 4. Materials and Methods

Oral swab samples from mink infected with SARS-CoV-2 were obtained from national surveillance monitoring of commercial farms in Poland during 2021–2023. They were collected by swabbing the oral cavity of mink with Copan Universal transport (UTM-RT) System (Copan, Brescia, Italy) and kept at 4 °C until delivery to the laboratory.

**Figure 6:**
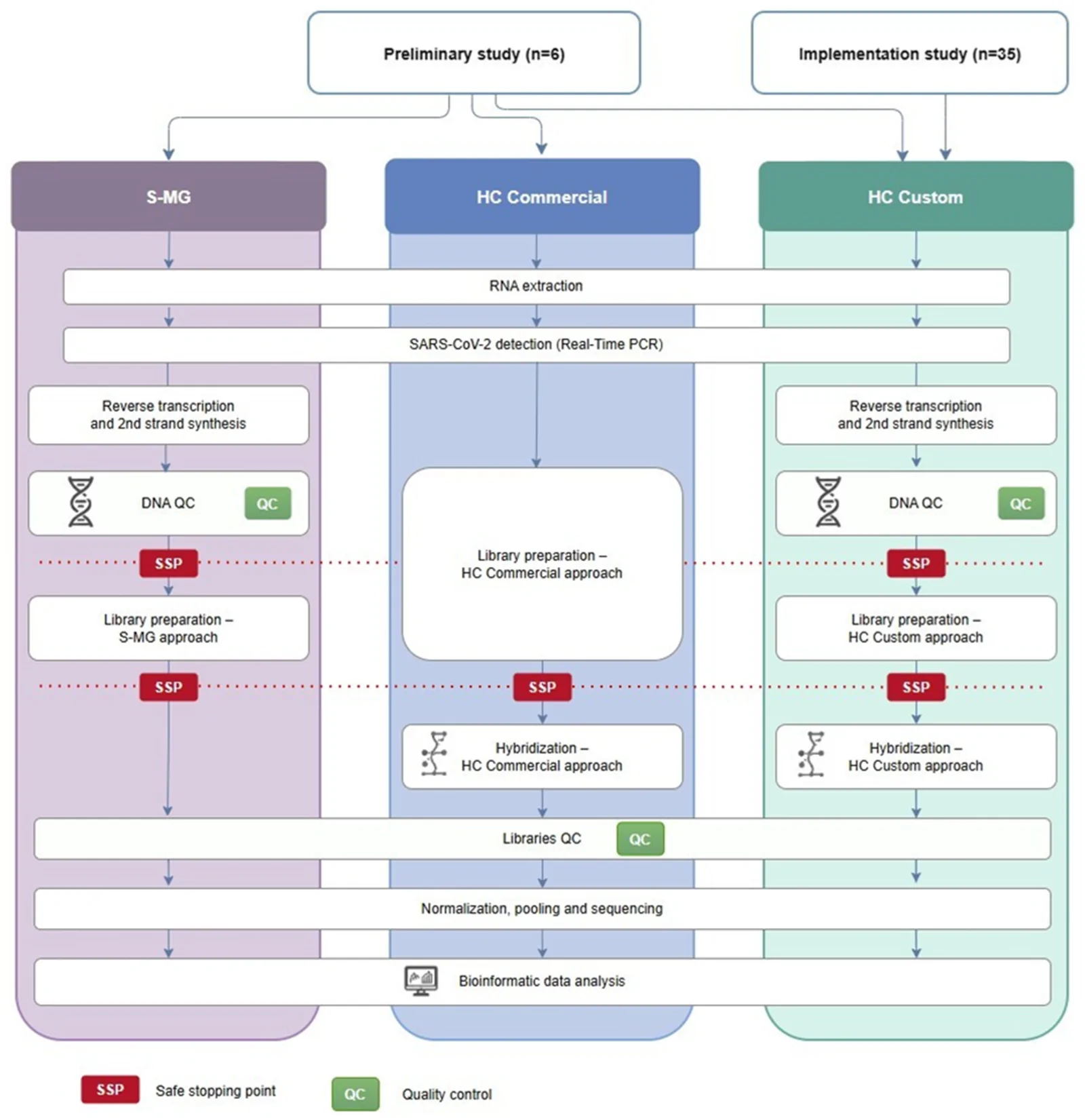
Scheme for the lab work on SARS-CoV-2 sequencing for the three tested methods.

For a comparative study, six samples (S1–S6) were prepared for high-throughput sequencing using three different library preparation protocols: (i) a shotgun metagenomic approach with KAPA HyperPlus by Roche(S-MG), (ii) a probe-based enrichment protocol using KAPA RNA HyperCap Workflow v1.0 by Roche(HC Commercial), and (iii) a custom probe-based method (HC Custom). Additionally, 35 samples were processed during the implementation study of the HC Custom method. The scheme for lab work across the three tested methods is shown in Figure 6.

### 4.1. RNA Extraction

Total nucleic acid extraction was performed from 200 µL of the mink oral swabs using an IndiMag Pathogen Kit (Indical, Leipzig, Germany) and an IndiMag 48s instrument for automated nucleic acid extraction, following the manufacturer’s protocol. Nucleic acids were eluted in 100 μL of AVE buffer. Extracted RNA was immediately used for detection, reverse transcription, and downstream sequencing.

### 4.2. SARS-CoV-2 Detection

Testing for SARS-CoV-2 was done using the real-time RT-PCR method previously described by Corman et al. [30]. This method targeted the E gene of sarbecoviruses, as well as N and RdRp gene fragments specific for SARS-CoV-2. Samples with Ct values of up to 36 were classified as SARS-CoV-2 positive and subjected to sequencing.

### 4.3. Reverse Transcription and Second-Strand Synthesis

To generate cDNA from RNA, reverse transcription (RT) was performed using the SuperScript IV First Strand Synthesis kit (Invitrogen (Carlsbad, CA, USA), Thermo Fisher Scientific (Waltham, MA, USA)), with the maximum RNA volume of 44 µL. Complementary strand synthesis was performed by adding DNA polymerase I (Klenow Fragment) (New England Biolabs (Ipswich, MA, USA)) to the mixture at a 1:42 volume ratio, incubating at 37 °C for 60 min, then at 75 °C for 10 min, and storing at 4 °C. The reaction was purified using KAPA Pure Beads (Roche, Basel, Switzerland) at a 0.8× (beads/sample) ratio.

### 4.4. Quality Control of dsDNA

Double-stranded DNA (dsDNA) was both quality- and quantity-controlled. Quality measurements were performed using a NanoDrop One (Thermo Fisher Scientific) spectrophotometer; the integrity of the genetic material was assessed using capillary electrophoresis on a Fragment Analyzer instrument (Agilent Technologies) and the DNF-468 HS genomic DNA 50Kb Analysis Kit (Agilent Technologies). Quantity measurements were carried out using the Qubit 3.0 instrument (Thermo Fisher Scientific) and the Qubit dsDNA HS Assay Kit. The dsDNA was stored at −20 °C (safe stopping point) until library preparation for HC Custom and S-MG.

### 4.5. Library Preparation

#### 4.5.1. Shotgun Metagenomic Approach (S-MG)

Libraries were prepared using the KAPA HyperPlus Kit and KAPA UDI adapters according to the Roche manufacturer’s instructions. Up to 100 ng of input dsDNA was used when available, while low-yield samples (a common problem with mink clinical swabs) were processed with 25 ng of input DNA. Libraries were amplified by PCR for 8 cycles.

#### 4.5.2. Commercial Probe-Based Approach (HC Commercial)

Libraries were prepared using the KAPA RNA HyperCap Workflow v1.0 following the manufacturer’s recommendations. Up to 10 µL of RNA was used directly as input for library preparation. The parameters for RNA thermo-fragmentation were set to 1 min at 65 °C. For library amplification, 19 PCR cycles were used. The KAPA HyperCap SARS-CoV-2 probe panel (Roche) was used for virus enrichment. The libraries were amplified again after hybridization with an additional 18 PCR cycles.

#### 4.5.3. Custom Probe-Based Approach (HC Custom)

Libraries were prepared using the KAPA HyperPlus kit with KAPA UDI adapters. Due to the variability in the amount, quality, and integrity of dsDNA obtained from clinical mink samples, modifications were made. Three different input amounts: 25 ng, 50 ng, and 100 ng of dsDNA were used, depending on the yield of RT. Based on the initial dsDNA input, the number of PCR cycles during library amplification was adjusted as follows: 100 ng—8 cycles, 50 ng—9 cycles, and 25 ng—10 cycles.

The library preparation protocol was further adjusted to improve the integrity of the obtained dsDNA. Since more intact DNA requires a longer fragmentation time than degraded DNA, the fragmentation time was modified accordingly. For highly intact material (average size of genomic DNA fragments > 2000), a 12-min fragmentation time was used, while for partially degraded material (average fragment size < 2000), a 9-min fragmentation time was used. Highly degraded samples (barely visible or invisible smear on the electropherogram) were processed for 5 min.

Next, a double-size selection was performed using KAPA Pure Beads (first size cut: 0.4×; second size cut: 0.6×). This step was omitted for highly degraded samples to minimize the loss of genetic material.

The libraries were pooled for hybridization into groups of up to six samples at a ratio of 250 ng of sample library per 1 µL of probe solution. For pools comprising at least five samples, the procedure was performed according to the manufacturer’s protocol (KAPA RNA HyperCap Workflow v1.0 by Roche). The multiplexing was performed using samples with similar viral content, as indicated by Ct values.

Pooled libraries were hybridized following Sections 5–7 of the KAPA RNA HyperCap Workflow v1.0 protocol (using 18 PCR cycles and the KAPA HyperCap SARS-CoV-2 probe panel (Roche). This process was conducted according to the manufacturer’s recommendations.

#### 4.5.4. Quality Control of the Libraries

Before sequencing, all amplified libraries prepared by each method were quality-assessed using a Fragment Analyzer (DNF-473 NGS Fragment Kit) and quantified with a Qubit 3.0 (Qubit dsDNA HS Assay Kit).

### 4.6. Normalization, Pooling, and Sequencing

Metagenomic libraries were normalized to equal molarity before sequencing. Libraries prepared using both hybrid capture approaches were normalized to equal molarity before hybridization.

Metagenomic samples were sequenced to an expected data output of 10 GB per sample. For both hybrid capture approaches, six samples from the preliminary study were sequenced at approximately 1 GB per sample. After assessing data from all approaches during the comparative stage of the study, the expected output was further reduced to 0.5 GB per sample for the implementation stage to avoid redundant data. Libraries were sequenced in paired-end mode on the Illumina platforms (MiSeq; 2 × 300 bp or NextSeq; 2 × 150 bp (Illumina, San Diego, CA, USA)).

### 4.7. Bioinformatic Data Analysis

Quality assessment of raw reads was performed using FastQC software (v0.11.9). Adapter trimming and quality filtering were conducted with fastp software (v0.23.2) [31]. The processed reads were aligned to the reference genome MN996528.1 using BWA-MEM [32]. Variant calling was performed with lofreq (v2.1.5) [33], and the resulting calls were normalized and filtered using bcftools, retaining only variants with a variant allele frequency (VAF) > 75% and a read depth (DP) > 10×. High-confidence variants were incorporated into the reference sequence using bcftools consensus [34] to generate consensus sequences. A single round of polishing was subsequently performed on the consensus sequences using Pilon (v1.23) [35] solely to identify large structural variants. Alignment statistics were calculated using SAMtools [34]. Data visualization was performed in R with the ggplot2 package [36]. Clade assignment and sequence quality control were conducted using Nextclade [37] and the Pangolin lineage classification tool [38]. The detailed comparison of sequences, including the identification of ambiguous positions (N) from the preliminary study, was performed using Jalview (v2.11.5.1) [39].

The parameters considered in the study included: mapping rate, depth of coverage, breadth and uniformity of coverage, and mean mapq. The mapping rate is the ratio of reads mapped to the SARS-CoV-2 genome to the total reads [40]. The depth of coverage presents the average sequencing depth per sample. The breadth-of-coverage assessment helps identify potential gaps or regions of low depth in the viral sequence [17]. A uniform depth characterizes a consistent depth across the sequence, without under- or oversequenced regions, which can disturb variant calling or cause some regions to have higher depth at the expense of others [17]. Mean mapq is a measure of the average mapping quality of mapped reads; high scores indicate high confidence in correctly aligned reads. In BWA-MEM, the mapping quality of a unique read is set to 60 [41].

### 4.8. Statistics

Statistical analyses were performed in R software (v4.5.1), and data visualizations were generated using the ggplot2 package (v4.0.0). For the comparative study (*n* = 6 samples), differences among sequencing methods with respect to average depth of coverage, mapping rate, and breadth of coverage were assessed using the Friedman rank-sum test. Significant results were further investigated using pairwise Wilcoxon signed-rank tests with Benjamini–Hochberg correction for multiple testing.

For the implementation study (*n* = 35 samples), the relationship between cycle threshold (Ct) values and sequencing performance metrics, including average depth of coverage, mapping rate, and breadth of coverage, was evaluated using Spearman’s rank correlation coefficient (ρ). This non-parametric approach was selected due to the distribution and characteristics of the analyzed variables. The 95% confidence intervals for Spearman’s correlation coefficients were estimated using bootstrap resampling of paired observations. To account for multiple comparisons, *p*-values were adjusted using the Benjamini–Hochberg procedure. Correlation coefficients and adjusted *p*-values were reported. Statistical significance was defined as an adjusted *p*-value < 0.05.

## 5. Conclusions

All three of the compared approaches produced high-quality results suitable for the identification of SARS-CoV-2 variants. Among them, the advantages of HC Custom are provided to support its implementation in the standard laboratory protocol. Compared with the S-MG approach, HC Custom exhibited markedly higher sensitivity and fewer ambiguous bases at the sequence ends, while maintaining lower costs. Compared with the HC Commercial method, HC Custom tends to generate higher-quality sequences, but the differences were not statistically significant. However, HC Custom is more sensitive than HC Commercial. It allows adjustment of the library preparation process according to both the sample amount and its integrity; thus, it can be useful for low-input and degraded material, like in mink-origin samples. However, it requires more time than HC Commercial and substantially more hands-on work than S-MG. Another limitation of the custom method is that it cannot sequence samples with Ct values higher than 33.9, and we did not evaluate its ability to detect low-frequency variants.

To the best of our knowledge, this is the first study comparing different SARS-CoV-2 sequencing approaches applied to clinical mink samples. Our findings demonstrate that evaluating each sample individually and adapting HTS protocol parameters can improve the performance of SARS-CoV-2 whole-genome sequencing. This study provides a useful tool for monitoring SARS-CoV-2 variants in mink. It may also support the optimization of viral sequencing from challenging or degraded samples, including DNA viruses, not only from mink but also from other sources.

## Figures and Tables

**Table 1:** Sequence quality and variants classification of the tested samples prepared by different methods.

Sample ID	Ct Ranges	Sequence ID	Method	Mapping Rate (%)	Breadth of Coverage (%)	Average Depth of Coverage (×)	Mean Mapq	Clade	Nextclade_pango
S1	19–21	230314_6835	S-MG	0.05	100.00	69	37.6	20B	B.1.1.44
		210924_4931	HC Commercial	96.50	100.00	16,102	60	20B	B.1.1.44
		230428_7083	HC Custom	90.88	100.00	2771	60	20B	B.1.1.44
S2	24–28	211123_5130 *	*S-MG*	*0.003*	*97.13*	*9*	*30.3*	*20B*	*B.1.1.44*
		210924_4932	HC Commercial	46.91	99.90	508	60	20B	B.1.1.44
		230324_6905	HC Custom	78.50	100.00	30,974	59.9	20B	B.1.1.44
S3	16–17	211208_5140	S-MG	0.14	100.00	440	53.6	20B	B.1.1.44
		210924_4934	HC Commercial	98.18	100.00	42,736	60	20B	B.1.1.44
		230428_7084	HC Custom	99.40	100.00	93,654	60	20B	B.1.1.44
S4	15–19	211123_5113	S-MG	0.08	100.00	61	48.4	20B	B.1.1.44
		210924_4938	HC Commercial	98.35	100.00	30,840	60	20B	B.1.1.44
		230428_7085	HC Custom	99.11	100.00	115,846	60	20B	B.1.1.44
S5	20	240312_9433	S-MG	0.02	99.90	51	59.3	22B	BA.5.2
		240301_9389	HC Commercial	82.95	99.997	26,647	60	22B	BA.5.2
		240301_9391	HC Custom	98.10	100.00	11,588	60	22B	BA.5.2
S6	21	240319_9458	S-MG	0.35	100.00	932	58.6	22B	BA.5.2
		240301_9390	HC Commercial	81.64	99.99	36,558	60	22B	BA.5.2
		240301_9392	HC Custom	97.99	100.00	91,768	60	22B	BA.5.2

* The sequence in italics is under the quality standards.

**Table 2:** Comparison of practical aspects and efficiency of S-MG, HC Commercial, and HC Custom.

	S-MG	HC Commercial	HC Custom
Laboratory workflow time efficiency	++ *	+	+
Cost efficiency	+	+++	+++
Sample-dependent adaptability	+	+	+++
Bioinformatics workflow efficiency	+	++	++
Quality control points	++	+	++

* The number of plus signs indicates the relative performance of each method for a given criterion, with more plus signs indicating better performance (+ low, ++ moderate, +++ high).

## Data Availability

The data that support the findings of this study are openly available in the NCBI Sequence Read Archive (SRA) under BioProject accession number PRJNA1462178.

## Supplementary Materials

The following supporting information can be downloaded at https://www.mdpi.com/article/10.3390/ijms27219638/s1.

## Abbreviations

The following abbreviations are used in this manuscript:

SARS-CoV-2	Severe acute respiratory syndrome coronavirus 2
VUM	Variants Under Monitoring
VOI	Variants of Interest
VOC	Variants of Concern
WHO	World Health Organization
ECDC	European Centre for Disease Prevention and Control
HTS	High-throughput sequencing
HC Commercial	Commercial hybrid-capture sequencing
HC Custom	Custom hybrid-capture sequencing
S-MG	Shotgun metagenomic sequencing
SRA	Sequence Read Archive
NCBI	National Center for Biotechnology Information
N	Ambiguous nucleotides
RT	Reverse transcription
dsDNA	Double-stranded DNA
